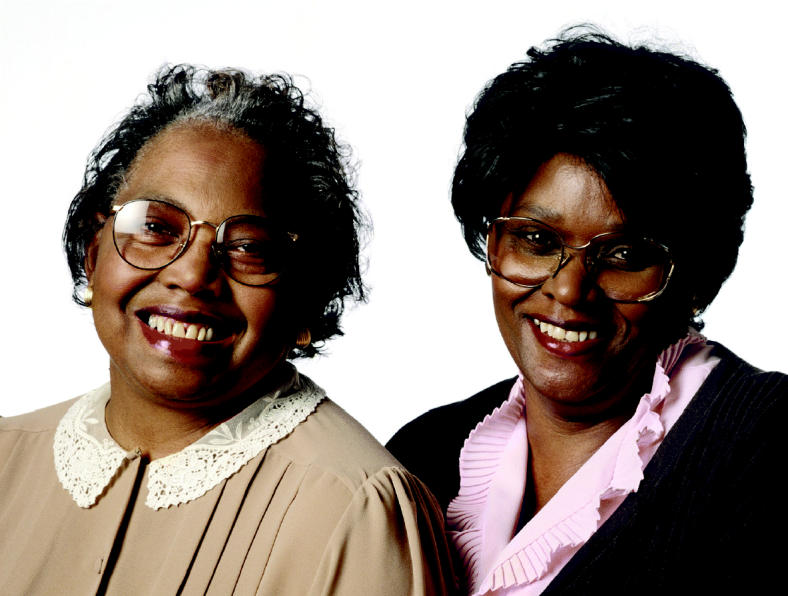# Headliners: Breast Cancer: Repair of DNA Damage Differs Between Sisters With and Without Breast Cancer

**Published:** 2005-05

**Authors:** Jerry Phelps

Kennedy DO, Agrawal M, Shen J, Terry MB, Zhang FF, Senie RT, Motykiewicz G, Santella RM. 2005. DNA repair capacity of lymphoblastoid cell lines from sisters discordant for breast cancer. J Natl Cancer Inst 97:127–132.

Like many types of cancer, breast cancer results from complex interactions of genetic predisposition and environmental exposures. Individuals differ in both their unique genetic makeup and the exposures that they encounter throughout their lifetimes. Damage to DNA is known to be a critical early step in the development of cancer; unrepaired damage leads to alterations in cellular functions. Therefore, individual differences in DNA repair capacity may influence the risk of developing cancer.

NIEHS grantee Regina M. Santella and colleagues at Columbia University in New York recently tested this hypothesis by analyzing DNA repair capacity in pairs of sisters, one of whom had breast cancer and one of whom did not. The researchers used cell culture lines for 158 breast cancer patients and 154 control sisters obtained from the Metropolitan New York Registry of Breast Cancer Families.

The cell cultures were treated with benzo[*a*]pyrene diolepoxide (BPDE), a DNA-damaging agent, then either harvested immediately or washed and cultured for four hours to allow DNA repair. The team then measured the number of BPDE–DNA adducts to determine the extent of DNA damage and repair capacity.

The DNA repair capacity of the breast cancer patients proved to be significantly lower than that of their sisters without breast cancer. When the Columbia researchers divided DNA repair capacity into quartiles, they observed a threefold higher cancer risk among the women with the lowest repair capacity compared with those women with the highest capacity.

This study supports the theory that deficits in DNA repair capacity are associated with higher susceptibility of breast cancer development. The finding may point to a valuable marker to identify women who are at high risk for the disease, especially among families with high incidence of breast cancer. Further research is necessary to determine if therapeutic interventions could improve DNA repair capacity.

## Figures and Tables

**Figure:**